# Salinity tolerance in Australian wild *Oryza* species varies widely and matches that observed in *O. sativa*

**DOI:** 10.1186/s12284-018-0257-7

**Published:** 2018-12-22

**Authors:** Yoav Yichie, Chris Brien, Bettina Berger, Thomas H. Roberts, Brian J. Atwell

**Affiliations:** 1 Sydney Institute of Agriculture, University of Sydney, Sydney, Australia; 2 School of Agriculture Food and Wine, University of Adelaide, Adelaide, Australia; 3 ​Australian Plant Phenomics Facility, The Plant Accelerator, Waite Research Institute, University of Adelaide, Adelaide, Australia; 4 Department of Biological Sciences, Macquarie University, Sydney, Australia

**Keywords:** *Oryza sativa*, *Oryza australiensis*, *Oryza meridionalis*, Salt, Australian native rice

## Abstract

**Background:**

Soil salinity is widespread in rice-producing areas globally, restricting both vegetative growth and grain yield. Attempts to improve the salt tolerance of Asian rice, *Oryza sativa*—the most salt sensitive of the major cereal crops—have met with limited success, due to the complexity of the trait and finite variation in salt responses among *O. sativa* lines. Naturally occurring variation among the more than 20 wild species of the *Oryza* genus has great potential to provide breeders with novel genes to improve resistance to salt. Here, through two distinct screening experiments, we investigated variation in salinity tolerance among accessions of two wild rice species endemic to Australia, *O. meridionalis* and *O. australiensis*, with *O. sativa* cultivars Pokkali and IR29 providing salt-tolerant and sensitive controls, respectively.

**Results:**

Rice plants were grown on soil supplemented with field-relevant concentrations of NaCl (0, 40, 80, and 100 mM) for 30 d, a period sufficient to reveal differences in growth and physiological traits. Two complementary screening approaches were used: destructive phenotyping and high-throughput image-based phenotyping. All genotypes displayed clear responses to salt treatment. In the first experiment, both salt-tolerant Pokkali and an *O. australiensis* accession (*Oa*-VR) showed the least reduction in biomass accumulation, SES score and chlorophyll content in response to salinity. Average shoot Na^+^/K^+^ values of these plants were the lowest among the genotypes tested. In the second experiment, plant responses to different levels of salt stress were quantified over time based on projected shoot area calculated from visible red-green-blue (RGB) and fluorescence images. Pokkali grew significantly faster than the other genotypes. Pokkali and *Oa*-VR plants displayed the same absolute growth rate under 80 and 100 mM, while *Oa*-D grew significantly slower with the same treatments. *Oa*-VR showed substantially less inhibition of growth in response to salinity when compared with *Oa*-D. Senescence was seen in *Oa*-D after 30 d treatment with 40 mM NaCl, while the putatively salt-tolerant *Oa*-VR had only minor leaf damage, even at higher salt treatments, with less than a 40% increase in relative senescence at 100 mM NaCl compared to 120% for *Oa*-VR.

**Conclusion:**

The combination of our two screening experiments uncovered striking levels of salt tolerance diversity among the Australian wild rice accessions tested and enabled analysis of their growth responses to a range of salt levels. Our results validate image-based phenotyping as a valuable tool for quantitative measurement of plant responses to abiotic stresses. They also highlight the potential of exotic germplasm to provide new genetic variation for salinity tolerance in rice.

**Electronic supplementary material:**

The online version of this article (10.1186/s12284-018-0257-7) contains supplementary material, which is available to authorized users.

## Introduction

Salinity, drought and heat are major abiotic stresses limiting the productivity of crop plants. Accumulation of toxic levels of salt as well as osmotic stress constitute a major threat to rice production worldwide, particularly in coastal rice-growing regions. Modern rice hybrids are some of the most salt-sensitive genotypes (Grattan et al. 2002; Munns et al. 2008; Qadir et al. 2014), with yield reductions evident above 30 mM NaCl (Ismail and Horie 2017) and survival of salt-sensitive genotypes compromised at 70 mM NaCl (Yeo et al. 1990). Rice is particularly vulnerable to salinity during the early seedling and reproductive stages (Zeng et al. 2002). The impact of salinity will be further exacerbated by factors such as marine inundation (Takagi et al. 2015). This has vast implications for food security because rice is the staple for much of Asia (Khush 2005) and throughout pantropical countries.

The basis of salt tolerance is polygenic, determined by a complex network of interactions involving signalling, stress-induced gene expression and membrane transporters (Wang et al. 2003). This complexity has complicated the search for physiological salt tolerance in rice because genotypes with tolerance in one trait are often intolerant in another (Yeo et al. 1990). Moreover, different developmental phases are characterised by distinct salt-tolerance mechanisms (Munns and Tester 2008), requiring breeding for genotypes with a suite of morphological, physiological and metabolic responses. Attempts to improve the salt tolerance of *O. sativa* have met with limited success due to these complexities as well as the interaction with nutritional factors, heterogeneity of field sites and other environmental factors such as heat and periodic drought (Flowers 2004; Yeo et al. 1990). Notwithstanding, the improvement of salt tolerance of rice at the seedling stage is a major breeding goal in many Asian countries, where seedlings must often establish in soils already contaminated by salt. While other crops might be better suited to salt-affected soils, few are suitable alternatives to rice because of its unique ability to grow when flooded.

Even though *O. sativa* represents less than 20% of the genetic diversity that exists in the 27 *Oryza* species (Zhu et al. 2007; Stein et al. 2018), there is still substantial variability in the tolerance to NaCl within this species (Gregorio et al. 1993; Lutts et al. 1995; Munns et al. 2016). In *O. sativa*, transport of Na^+^ to the shoot is a major determinant of salt tolerance (Yeo et al. 1987; Yadav et al. 1996; Ochiai et al. 2002). The activity of a vacuolar antiporter was found to increase salt tolerance (Fukuda et al. 2004). More recently, a novel quantitative trait locus (QTL) named *Saltol* was found to encode a trans-membrane protein, OsHKT1;5, which regulates K^+^/Na^+^ homeostasis under salt stress, increasing tolerance to salt (Ren et al. 2005; Thomson et al. 2010). Additional studies have identified other QTL and mutations for salt tolerance within *O. sativa* (Lang et al. 2001; Yao et al. 2005; Sabouri et al. 2008; Islam et al. 2011; Takagi et al. 2015) but the mechanisms of the proteins encoded in these loci are yet to be revealed.

The diversity of wild rice relatives would suggest that a novel salt-tolerance mechanism for rice breeding programs should come from the examination of *Oryza* species from natural populations, of which four are indigenous to Australia: *O. meridionalis, O. officinalis, O. rufipogon* and *O. australiensis* (Henry et al. 2010; Atwell et al. 2014). While the best evidence thus far for the ability of *Oryza* species to contribute stress-tolerance genes is the case of resistance to brown leaf hopper (Khush 1997; Rahman et al. 2009), abiotic factors have been powerful selective forces on these species in northern Australia, encouraging our search for tolerance to physical constraints on growth. For example, *O. meridionalis* and *O. australiensis* have superior heat tolerance compared with *O. sativa* (Scafaro et al. 2010) with the wild allelic form of the Rubisco activase gene responsible for this trait in *O. australiensis* (Scafaro et al. 2016).

Although the Australian endemic rices are poorly characterised, trials demonstrate the potential of using wild rice species introgressions to enhance the growth of *O. sativa* (Ballini et al. 2007). A recent study showed that Australia may be a centre of origin and segregation of the AA genome of *Oryza* and underlined the wide genetic diversity within the *Oryza* species that share this genome (Brozynska et al. 2016). Further diversity could be expected in the phylogenetic outlier *O. australiensis*, which is the sole species with an EE genome (Jacquemin et al. 2013). The discovery of many domesticated alleles within the wild species reinforces the hypothesis that wild relatives are a key asset for crop improvement (Brozynska et al. 2016).

Over recent years, several studies in cereals and legumes have utilised high-throughput phenotyping technology under controlled environments to gain a better understanding of the genetic architecture and the physiological processes associated with salinity stress (Hairmansis et al. 2014; Campbell et al. 2015, 2017; Atieno et al. 2017). However, this approach had not been applied to crop wild relatives. In a large-scale, non-destructive phenotyping facility (‘The Plant Accelerator’) we assembled shoot images of *O. sativa*, *O. meridionalis* and *O. australiensis* exposed to a range of salt treatments for five weeks during the early vegetative stage. We sought to examine developmentally specific salinity responses, growth dynamics and the complex relationship between different traits under salt stress in Australian wild rices pre-selected for inherent tolerance to salinity. Comparisons were made between these genotypes and *O. sativa* genotypes Pokkali (salt-tolerant) and IR29 (salt-sensitive). The broader context of this work was to gain insights into abiotic stress tolerance of exotic Australian genotypes with the aim of identifying key genes in subsequent research.

## Material and methods

### Plant material, growth conditions and salt treatments

#### Experiment 1

Five wild accessions chosen from two Australian endemic wild rice species, *O. meridionalis* and *O. australiensis*, were tested along with two cultivated varieties of *O. sativa*, Pokkali and IR29. The wild accessions were selected from a wide range of sites, including transiently saline waterways in the north and northwest of Australia. Approximately 30 genotypes were screened for symptoms and survival in preliminary experiments (unpublished data), exhibiting a wide spectrum of tolerance to 25–100 mM NaCl over a four-week treatment.

The initial testing led to a narrower selection of genotypes screened at Macquarie University, Sydney, Australia (lat. 33.7° S, long. 151.1° E) in spring 2016. Seeds were de-hulled and surface-sterilised by successive immersion in water (30 min), 4% commercial bleach (30 min) and at least five rinses with diH_2_O. Seedlings were then germinated in petri dishes in the dark at 28 °C (*O. sativa*) and 36 °C (wild rice) and grown for a further 5 d at 28 °C. After 8 d, two to four seedlings per genotype were sown in a 1.5-L polyvinyl chloride (PVC) pot (with drainage holes) containing 1.3 L of locally sourced clay-loam, slow-release fertiliser (Nutricote Standard Blue, Yates, 0.04%) and placed in the greenhouse. Seedlings were thinned, leaving one uniformly sized and healthy seedling in each pot 15 d after transplanting (DAT).

Salt treatments were applied to the top of the pots gradually in three stages from 25 DAT (25, up to 40 and up to 80 mM daily increments). The final NaCl concentrations for the first screening were 0, 40 and 80 mM NaCl—a total electrolyte concentration resulting in an electrical conductivity (EC) of 0.0, 0.5, 4.5, and 8.7 dS m^− 1^, respectively. Plants were watered once a day with ~ 50 mL per pot of their respective salt concentration (including 0.4 g L^− 1^ of Aquasol Soluble Fertiliser, Yates). A square aluminum tray was placed under each set of treatment pots and the drainage was collected every 3 d. Plants were exposed to salt treatments for 30 d in a controlled greenhouse with 30 °C/22 °C day/night temperature and relative humidity of 57% (± 9%, SD) during the day and 77% (± 2%, SD) at night.

A completely randomised design was used, with a minimum of five replicates (pots) for each plant genotype-treatment combination. The locations of the trays and of each pot within trays were changed randomly every 3 d to subject each one of the plants to the same conditions and to prevent neighbour effects. A few IR29 plants dehydrated two weeks after exposure to salt (80 mM NaCl treatment) and were removed from the statistical analysis.

#### Experiment 2

Seven lines of rice, including two cultivated *O. sativa* controls—Pokkali, a positive control (salt tolerant) and IR29, a negative control (salt sensitive)—were investigated at the four salt concentrations described above, with an additional salt treatment of 100 mM (EC = 10.5 dS m^− 1^). This experiment was performed in the South East Smarthouse at The Plant Accelerator (Australian Plant Phenomics Facility, University of Adelaide, Adelaide, Australia; lat. 34.9° S, long. 138.6° E) in the summer of 2017. The same greenhouse conditions and treatments were applied as in Experiment 1. The seedlings were sown and thinned following the same protocol as used in Experiment 1 in 2.5-L pots with 2.0–2.2 L of UC Davis-mix (2.5 g L^− 1^ Mini Osmocote® 16–3-9 + te) and the surface was covered with white gravel (particle size ~ 2–5 mm) to minimise evaporation from the pot and to reduce algal growth. For the first 7 DAT, each pot was watered daily with ~ 100 mL from the top. The pots were placed on top of square containers (93 mm diameter, 50 mm height) to prevent water from spilling onto the conveyor system and to allow the drainage water to be collected.

Salt treatments were applied gradually in four steps from 22 DAT to the square container (25, up to 40, up to 80 and up to 100 mM daily increments). The holes in the pots allowed for the infiltration of salt solution into the soil through capillary action. The water level was maintained constant by weighing each plant and watering to a target volume of 600 mL. Daily imaging and watering were continued for 30 d after salt treatment until 30 d after salting (DAS). The same post-harvest parameters were measured as in Experiment 1.

Image-based high-throughput phenotyping was performed on rice genotypes selected from the wider group tested in initial screening experiment (spring 2016).

A split-unit design was performed concurrently, where 12 lanes × 14 positions (5–12, 15–20) with six replicates to assign the factorial set of treatments were occupied. Each replicate occupied two consecutive lanes and included all 28 rice line-treatment combinations. Each replicate comprised seven main units, each consisting of four carts arranged in a grid of two lanes × two positions. Thus, the 42 main units formed a grid of 6 reps × 7 main positions. The plant lines were assigned to main units using a 7 × 6 Youden square. The four salt treatments were assigned to the four carts within each main unit using a resolved incomplete block design for four treatments in blocks of size 2. The design was randomised using dae (Brien, 2018), a package for the R statistical computing environment (R Core Team, 2018).

### Phenotyping of physiological traits

#### Gas exchange values

Plants were phenotyped throughout the experiment for growth parameters. Gas exchange parameters such as photosynthesis, stomatal conductance and transpiration were measured on DAS 29 and DAS 30 (for the first and second experiments, respectively) with an infrared, open gas exchange system (LI-6400, LICOR Inc., Lincoln, NE, USA). All gas measurements were completed on the same day between 10:00 am and 12:30 pm and were made on the youngest fully-expanded leaf (YFL) of each rice plant.

#### Growth and yield components

Plants were characterised for phenotypic responses to salinity stress on 30 d after salt application (DAS), the plants were harvested, and the following post-harvest parameters were determined. Shoot fresh weight (SFW) was measured for each plant immediately after harvest, as well as number of tillers. Plant shoots were dried at 65 °C in a ventilated oven for 48 h to constant weight and shoot dry weight (SDW) was measured.

#### Leaf chlorophyll determination

The YFL was collected from each plant on the day of harvest (DAS30); leaves were flash-frozen in liquid nitrogen after being washed with diH_2_O. Chlorophyll was extracted using 95% ethanol and total chlorophyll was determined (Mackinney 1941). Chlorophyll concentrations at each salt level were normalised against control (non-salinised) levels.

#### Ion assay

The YFL of each plant was collected as described above. Samples were washed thoroughly and dried at 70 °C. Each sample was weighed and extracted with 10 ml 0.1 N acetic acid for every 10 mg of dried tissue. Samples were placed in a water bath at 90 °C for 3 h. Samples were diluted 10 times after the extracted tissues were cooled at room temperature. Sodium and potassium concentrations were measured using an Agilent 4200 Microwave Plasma Atomic Emission Spectrometer (Agilent Technologies, Melbourne, Australia).

#### Salinity tolerance estimation

Salinity tolerance (ST) was determined by the percentage ratio of mean shoot dry weight (80 mM NaCl) divided by mean shoot dry weight (no salt) [SDW (salt treatment))/ (SDW (control)) × 100]. Each plant was evaluated for seedling stage salinity tolerance based on visual symptoms using the International Rice Research Institute (IRRI) standard evaluation system (SES) scores (IRRI 2013).

### RGB/fluorescence image capture and image analysis

Two types of non-destructive imaging systems were utilised to address our questions: RGB (red-green-blue)/visible spectrum and fluorescence (FLUO). Standard RGB images had a resolution of 8 M pixels, while fluorescence images had a resolution of 5 M pixels (Berger et al. 2012). However, in our experiment, some plants attained a physical height exceeding that of the field of view of the RGB camera (the RGB camera was closer to the plants than the fluorescence camera). Thus, we chose to use the projected shoot area (PSA) based on RGB images at the beginning of the experiment (DAS 4–19) and PSA based on fluorescence at the end (DAS 20 onwards). For the RGB images, PSA is the sum of the areas as measured (in kilopixels) from two side views at an angular separation of 90 degrees and a view from above; for the fluorescent images, PSA is the sum of the areas as measured (in kilopixels) from two side views at an angular separation of 90 degrees.

Consequently, a hybrid PSA trait was calculated using the RGB images for DAS 4–19 and the FLUO images for DAS 20 onwards. The PSA data from the FLUO images were transformed using the linear relationship between PSA from the RGB images and PSA from the FLUO images (for DAS 20). The conversion was made on the raw observations and then the new data were prepared for each plant as described below. Water levels were monitored and adjusted daily by the Scanalyzer 3D weighing and watering system (LemnaTec GmbH, Aachen, Germany), with pot weight before and after watering being recorded.

To screen for osmotic tolerance, plant growth rate after the addition of NaCl was determined using the hybrid PSA trait from DAS 2 to 30, where DAS 0 corresponded to the commencement of the salt treatments to generate the PSA of the plant. The results of the high-throughput screening focused on PSA and the absolute growth rate (AGR) and relative growth rate (RGR) derived for these plants. The traits were obtained as described (Al-Tamimi et al. 2016). The PSA AGR and PSA RGR were calculated from the PSA values by determining the difference between consecutive PSA and ln(PSA) values, respectively, and dividing by the time difference. Similarly, the daily water loss from each pot was obtained by subtracting the weight before watering in the current imaging day from the weight after watering on the previous imaging day. The PSA water use index (WUI) was calculated daily by dividing the PSA AGR by the water use. On the one occasion that water use values were negative due to leakage from a storm, values were replaced with blank values to avoid affecting the smoothed spline curve fitting.

### Data preparation and statistical analysis

#### First experiment

Statistical significance of phenotypic traits was determined by Analysis of Variance (ANOVA) with Tukey HSD multiple comparison with significant values of *P* ≤ 0.05 and *P* ≤ 0.01. Pairwise comparisons were conducted using LSD-Test and Tukey adjustments to produce *p*-values for the significant differences of specific pairs using the R package ggplot2 (Wickham, 2009). A linear regression model was used to calculate the Salinity Tolerance (ST) against sodium and potassium concentrations and the corresponding r coefficients.

#### Second experiment

Data from the Smarthouse were first analysed using imageData (Brien, 2018) to determine subjectively the degree of smoothing required to produce growth curves using PSA values; this approach removed noise in the data while accurately capturing the underlying growth trajectories. PSA AGR and the PSA RGR were derived by fitting natural cubic smoothing splines to the data for each plant with different settings of the smoothing parameter degrees of freedom (df) (Al-Tamimi et al. 2016). A df value of five was chosen, as it gave the most satisfactory results over all three traits. The water use rate was also smoothed by fitting a spline using df = 5. After examination of the plots for the smoothed traits sPSA, sPSA AGR and sPSA RGR, we decided to investigate growth for six DAS endpoints (DAS 4, 9, 14, 19, 23 and 28) and thus the response of the rice plants to salt treatment was separated into five corresponding intervals.

Correlation analysis was performed on the biomass-related metrics (smoothed PSA 28 and 30 DAS) and manual measurements of SFW and SDW. Both SDW and SFW displayed a strong positive correlation with PSA, with the highest correlation between smoothed PSA and SDW (r^2^ = 0.966, *P* = 0.001, *n* = 168) (Additional file 1: Figure S1) using the squared Pearson correlation coefficient. A similar strong positive correlation was found (r^2^ = 0.96, *P* = 0.001, *n* = 72) in a previous study that measured the correlation between PSA and total plant area using a leaf area meter (LI-3100C; LI-COR) (Campbell et al. 2015). This validates our experimental set-up as suitable to monitor plant growth and physiological responses to salt treatments and indicates that PSA is an accurate and sensitive metric for assessing plant biomass accumulation in response to salinity.

To produce phenotypic means adjusted for the spatial variation in the Smarthouse, a mixed-model analysis was performed for each trait using the R package ASReml-R (Butler et al. 2009) and asremlPlus (Brien, 2018), both packages for the R statistical computing environment (R Core Team, 2018). The maximal mixed model used was described previously (Al-Tamimi et al. 2016).

Residual variances were tested using REML ratio tests with α = 0.05 to test whether the differences were significant for both salinities and lines, for just one of them, or not at all. In order to reflect the results of these tests and to check that the assumptions underlying the analysis were met, the model was modified to residual-versus-fitted value plots and normal probability plots of the residuals inspected. Wald F-tests were conducted to check whether an interaction (between lines and salinity) was significant, for its main effects. The predicted means and standard errors were obtained for the selected model for salinity and lines effects. To compare a pair of predicted means the *p*-value for an approximate t-test was calculate from the predicted means and their standard errors. However, for cases in which the variances were unequal, these were computed for each prediction using the average variance of the pairwise differences over all pairwise differences in which the prediction was involved and are only approximate.

## Results

### First screening (experiment 1)

After 30 d of growth in non-salinised (control) conditions, *O. sativa*, *O. meridionalis* and *O. australiensis* shoot dry biomass ranged from 11.5 (IR29) to 22 g (Pokkali), with the exception of *Oa*-KR for which dry biomass reached 34 g by the end of the experiment. Average chlorophyll concentrations ranged from 1.67 to 3.94 mg g^− 1^ (SDW), while mean net photosynthetic rates ranged from 14.9 to 19.9 μmol m^− 2^ s^− 1^ (Additional file 2: Table S1).

**Fig. 1:**
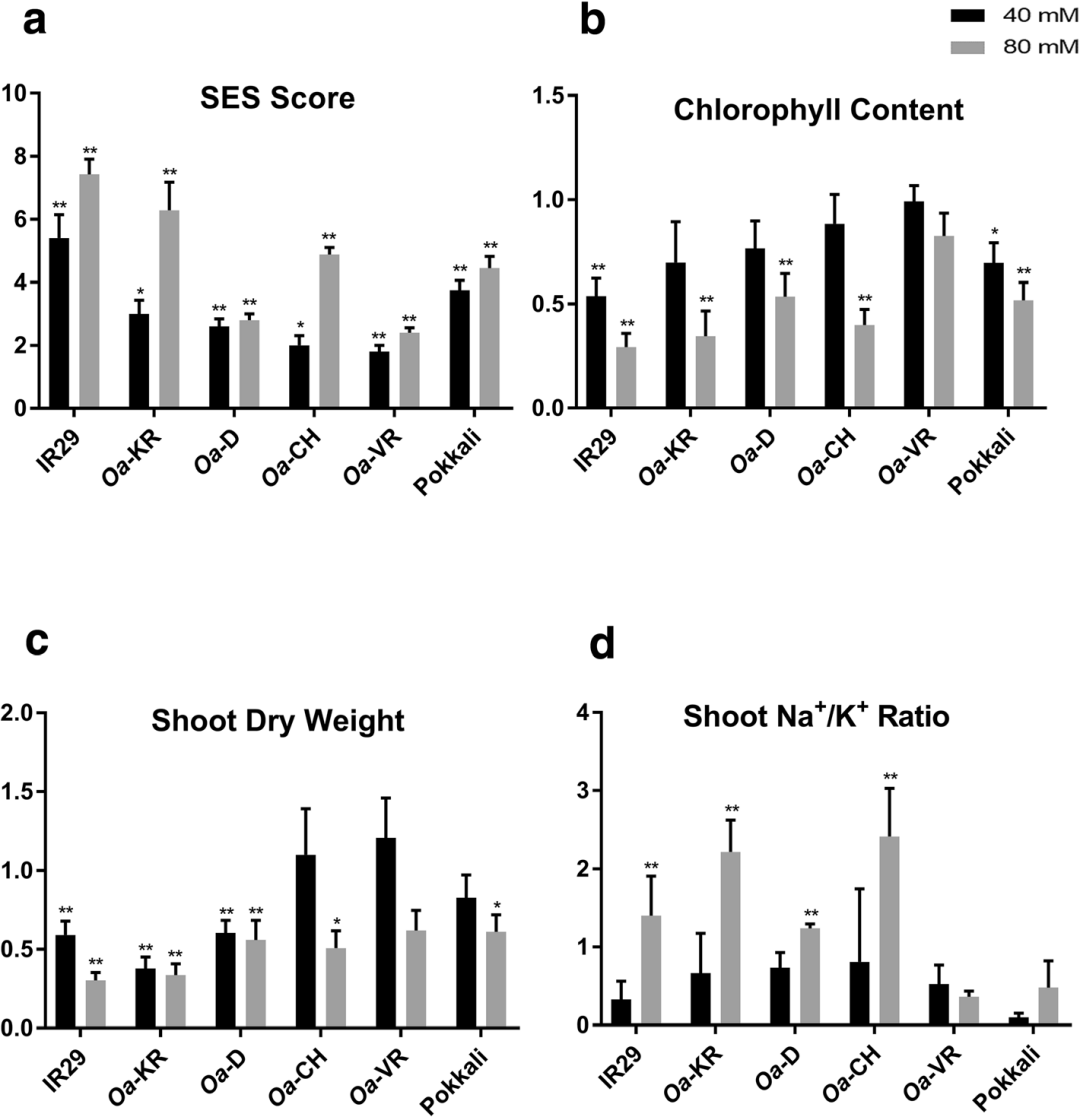
**a** Standard Evaluation System (SES) scores [1-9]; **b** Normalized chlorophyll content (as a ratio of the control); **c** Normalized biomass growth by SDW (as a ratio of the control) and **d** Shoot Na^+^/K^+^ ratio of the four wild *Oryza* accessions and *O. sativa* controls: IR29 (salt sensitive) and Pokkali (salt tolerant). Trait means (± standard errors) are shown for each genotype under 40 and 80 mM NaCl (EC = 8.7 dS m^-1^) at the seedling stage. For **a**, **b** and **c**, asterisks indicate significant differences from the non-salinised control for the same genotype, based on Student‘s t test (*P < 0.05, **P < 0.01). For **d**, asterisks indicate significant differences between 40 and 80 mM based on Student‘s t test (*P < 0.05, **P < 0.01) because the ratios (as used for **a** to **c**) were so low in non-salinised controls as to be negligible, whereas the increase in ratio from 40 to 80 mM was highly relevant salt tolerance differences between genotypes

Relative to the non-salinised control plants, clear differences in phenotype became apparent after exposure to 40 and 80 mM NaCl. Visual symptoms across all six genotypes were assessed by SES, showing salt-induced injury when expressed relative to control plants (for which SES = 1.0; i.e. no loss of leaf function). In the oldest leaves of IR29, SES reached 5.4 at 40 mM and 8.3 at 80 mM NaCl, reflecting loss of function in all but the most recently expanded leaves (Fig. 1a). In the most salt-tolerant genotype (*Oa*-VR), SES was 1.8 at 40 mM and 2.4 at 80 mM NaCl. Chlorophyll concentrations followed an identical pattern (Fig. 1b), where in the salt-sensitive genotype (IR29) there was a 34% reduction at 40 mM and a 72% reduction at 80 mM NaCl, while in *Oa*-VR there was no change in chlorophyll concentration at 40 mM and a 19% reduction at 80 mM NaCl.

Seedling fresh and dry biomass were measured 30 DAS. Because of inherent variation in the growth rate of the wild species, biomass of plants treated with 40 and 80 mM NaCl are shown relative to control plants (Fig. 1c - dry weights; Additional file 2: Table S1). There was no growth penalty in the two most tolerant wild rice genotypes (*Oa*-VR and *Oa*-CH) at 40 mM NaCl, with both being considerably more tolerant than the salt-tolerant *O. sativa* genotype, Pokkali. The most salt-sensitive wild rice line (*Oa*-D) was as susceptible to salt as IR29 at 40 mM NaCl. These data are consistent with visual symptoms, indicating that *Oa*-VR was the most salt-tolerant wild *Oryza* accession and *Oa*-D the least tolerant. Na:K ratio calculated at 40 and 80 mM NaCl (Fig. 1d) revealed the lowest Na:K ratios in *Oa*-VR and Pokkali while the other wild rice genotypes and IR29 had progressively higher ratios, reaching an average of 2.41 for *Oa*-CH.

**Fig. 2:**
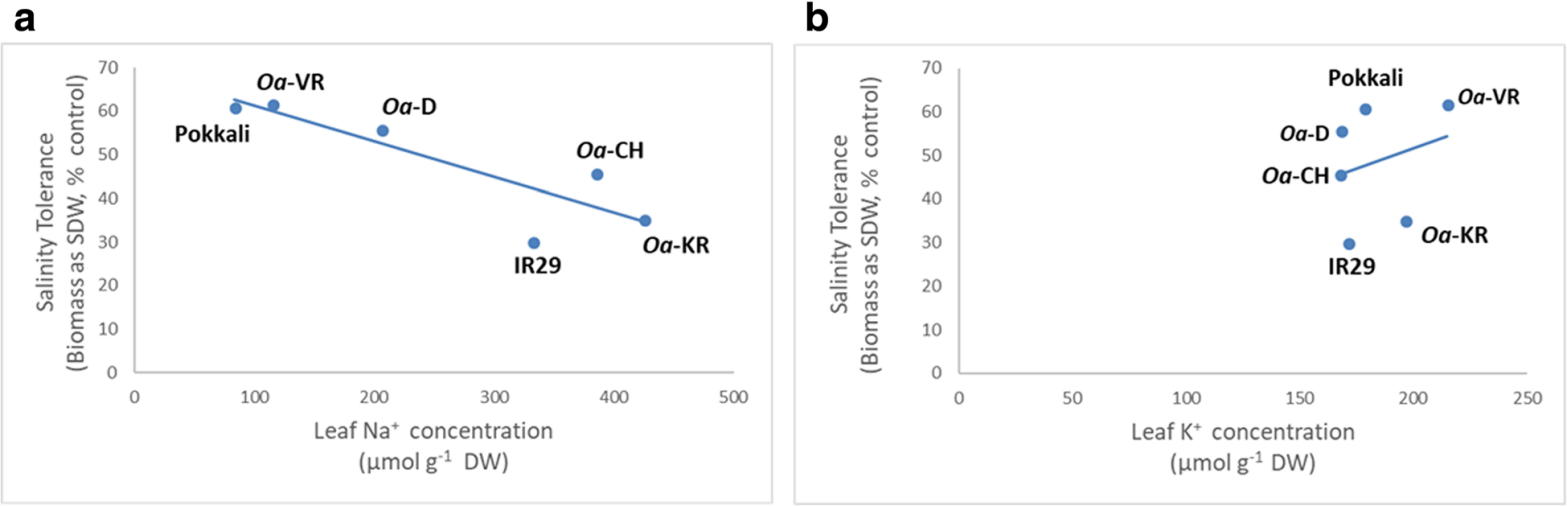
Linear regression of Salinity Tolerance (ST) against: **a** leaf Na^+^ concentrations [μmol Na^+^ g^-1^ (SDW)] (R^2^ = 0.75) and **b** leaf K^+^ concentrations [μmol Na^+^ g^-1^ (SDW)] (R^2^ = 0.69). ST was calculated as the percentage ratio of mean shoot dry weight (salt treatment, 80 mM of NaCl) divided by mean shoot dry weight (control, no salt) [SDW (salt treatment))/(SDW (control)) x 100]

Sodium and potassium ion concentrations were measured in the youngest fully expanded leaves, where tissues remained hydrated even in the salt-sensitive genotypes, as shown by the narrow range of variation in K^+^ concentrations (Fig. 2). The relationships between ion concentrations and leaf biomass (as a percentage of controls) illustrate the strong negative relationship between Na^+^ concentration and salinity tolerance, confirming that the exclusion of Na^+^ conferred physiological tolerance (Fig. 2). The three most salt-sensitive genotypes had 300–500 μmol Na^+^ g^− 1^ (SDW) while the most salt-tolerant genotypes had up to three times less Na^+^. A negative relationship between physiological tolerance (ST) and Na^+^ concentrations in the youngest fully expanded leaves was clear when all genotypes were compared (Fig. 2). A weak positive relationship was recorded between K^+^ concentrations in shoots and salinity tolerance. Notably, Na^+^ concentrations in *Oa*-VR and Pokkali were lowest of all six genotypes (114 and 83 μmol g^− 1^ (SDW), respectively) and when expressed on a tissue water basis (using the SFW/SDW ratio of 3.6 and 3.4, respectively) Na^+^ concentrations were 34 and 44 μmol g^− 1^ (FW), respectively; i.e. much lower than those in the soil solution in which they grew. *Oa*-VR accumulated 215 μmol K^+^ g^− 1^ (SDW), 20% more (*P* < 0.05) than the levels found in IR29 and *Oa*-D (171 and 168 μmol g^− 1^ (SDW), respectively).

Depending upon the genotype, ion toxicity symptoms were first visible in leaves 7–15 DAS. Initially, salt-induced symptoms were always restricted to the older leaves but increased progressively in severity and extent until only the most recently emerged leaves were unaffected (data not shown).

Measurements at 80 mM NaCl established that the negative effects of salt were consistent across three vegetative traits—plant height, SDW and number of tillers (Additional file 3: Table S2). Furthermore, damage measured by SES scores correlated negatively with these traits, as well as photosynthetic rates (*P* = 0.01).

### Plant accelerator (experiment 2)

**Fig. 3:**
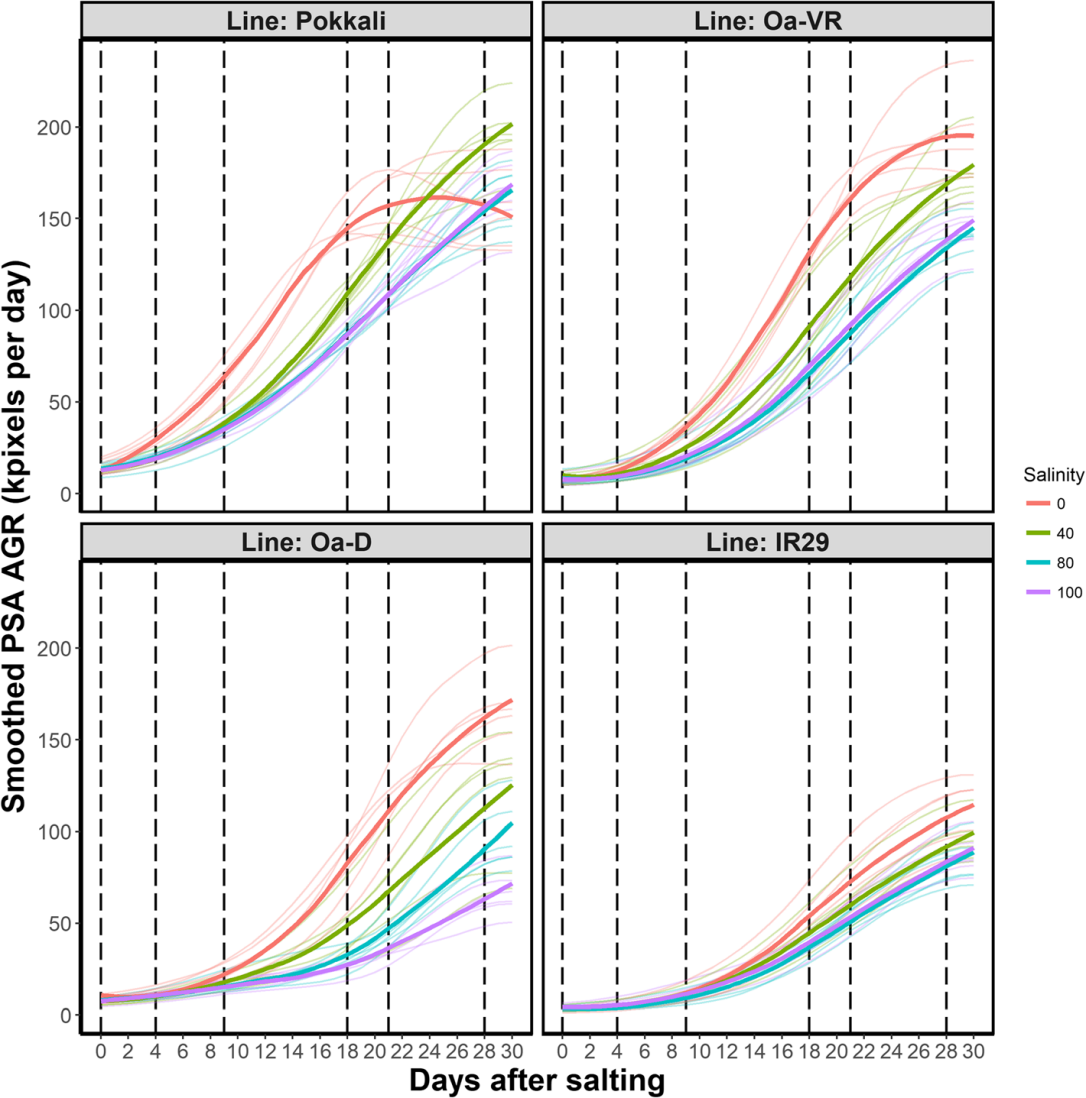
Absolute growth rates of Pokkali, *Oa*-VR, *Oa*-D and IR29 from 0 to 30 DAS including non-salinised controls. Smoothed AGR values were derived from projected shoot area (PSA) values to which splines had been fitted. Thin lines represent individual plants. Bold lines represent the grand average of the six replicates plants for each treatment. The vertical broken lines represent the tested intervals

There were no visual leaf symptoms or wilting in any genotype 4 d after salt was applied. Pokkali grew significantly faster (16.2 kpixels d^− 1^) than other lines over the first 9 d (*P* < 0.05) while IR29 grew slowest, in all treatments (Fig. 3; Additional file 4: Figure S2). The two wild rice species had the same relative growth rate at this earliest stage of salt treatment (*P* > 0.05), while Pokkali and IR29 grew significantly faster and slower, respectively (Additional file 5: Figure S3). Importantly, the average growth rates of the control plants during DAS 0 to 4 and 4 to 9 were significantly greater (*P* < 0.05) than any of the salt treatments (Fig. 3; Additional file 4: Figure S2). RGR in Pokkali declined steadily throughout the experiment, even in salt-treated plants (Additional file 4: Figure S2, Additional file 5: Figure S3), indicating that plants did not grow exponentially at any stage of the salt treatment. On the other hand, periods of exponential growth were observed in the other three genotypes, with exponential growth notably sustained in *Oa*-VR for the first 15 d of salt treatment (Additional file 5: Figure S3). After 23 DAS, RGR was lower (Pokkali, *Oa*-VR and *Oa*-D) or the same (IR29) in control plants when compared with salt-treated plants, which grew at 10% per day. These time-dependent shifts in the response of the genotypes to salinity were analysed using *p*-values for prediction mean differences within each interval identified in Fig. 3. While differential effects of salinity across genotypes were not seen in the absolute growth rate until plants had been exposed to salt for at least 19 d, salinity × genotype interactions were seen strongly in RGR from the beginning of the experiment. This is reflected in Additional file 5: Figure S3, where the changes in RGR in Pokkali plants reflected the vigorous canopy growth, early self-shading and distinctive, rapid canopy development rate compared with the other three genotypes tested.

There was a wide range of growth responses at each salt level in the seven genotypes imaged (Additional file 6: Figure S4), with IR29 notably the slowest growing genotype. Individual performances of the two *O. sativa* standard lines and two of the most contrasting *O. australiensis* accessions are represented at all four salt levels in Fig. 3. The reduction in shoot growth, as measured by PSA, was most pronounced at 80 and 100 mM NaCl, with smaller reductions at 40 mM NaCl (Fig. 3). By 12 DAS, non-salinised plants of all four genotypes were growing significantly faster than all salt-treated plants. Importantly, Pokkali, *Oa*-VR and *Oa*-D grew substantially faster than IR29: at 12 DAS, non-salinised control plants grew at 25.1, 13.8, 13.5 and 5.9 kilopixels d^− 1^ (as measured by PSA) in the four genotypes, respectively. Pokkali, *Oa*-VR and *Oa*-D treated with 100 mM NaCl were reduced to 78–88% of the controls, while no effect of 100 mM NaCl could be detected in IR29 plants. Despite the reputation of IR29 as a salt-sensitive genotype, its inherently slow growth made responses to NaCl difficult to detect in the early stages of vegetative development (Additional file 5: Figure S3). The divergence in AGR between plants grown at 80 and 100 mM NaCl was notable, with Pokkali and *Oa*-VR plants growing at the same rate in these two highest salt treatments, while *Oa*-D plants grew significantly slower at 100 mM than at 80 mM NaCl (Fig. 3). Importantly, *Oa*-VR showed substantially less inhibition of growth in response to salinity when compared with *Oa*-D, supporting the observation from the first experiment that *Oa*-VR is the most salt tolerant of the wild rice accessions tested (Fig. 3). The most severe reduction in PSA across all genotypes tested in the Plant Accelerator was an *O. meridionalis* genotype (*Om*-T), where there was a 27% reduction after DAS9 and a further reduction of almost 20% by DAS18 in 100 mM NaCl.

**Fig. 4:**
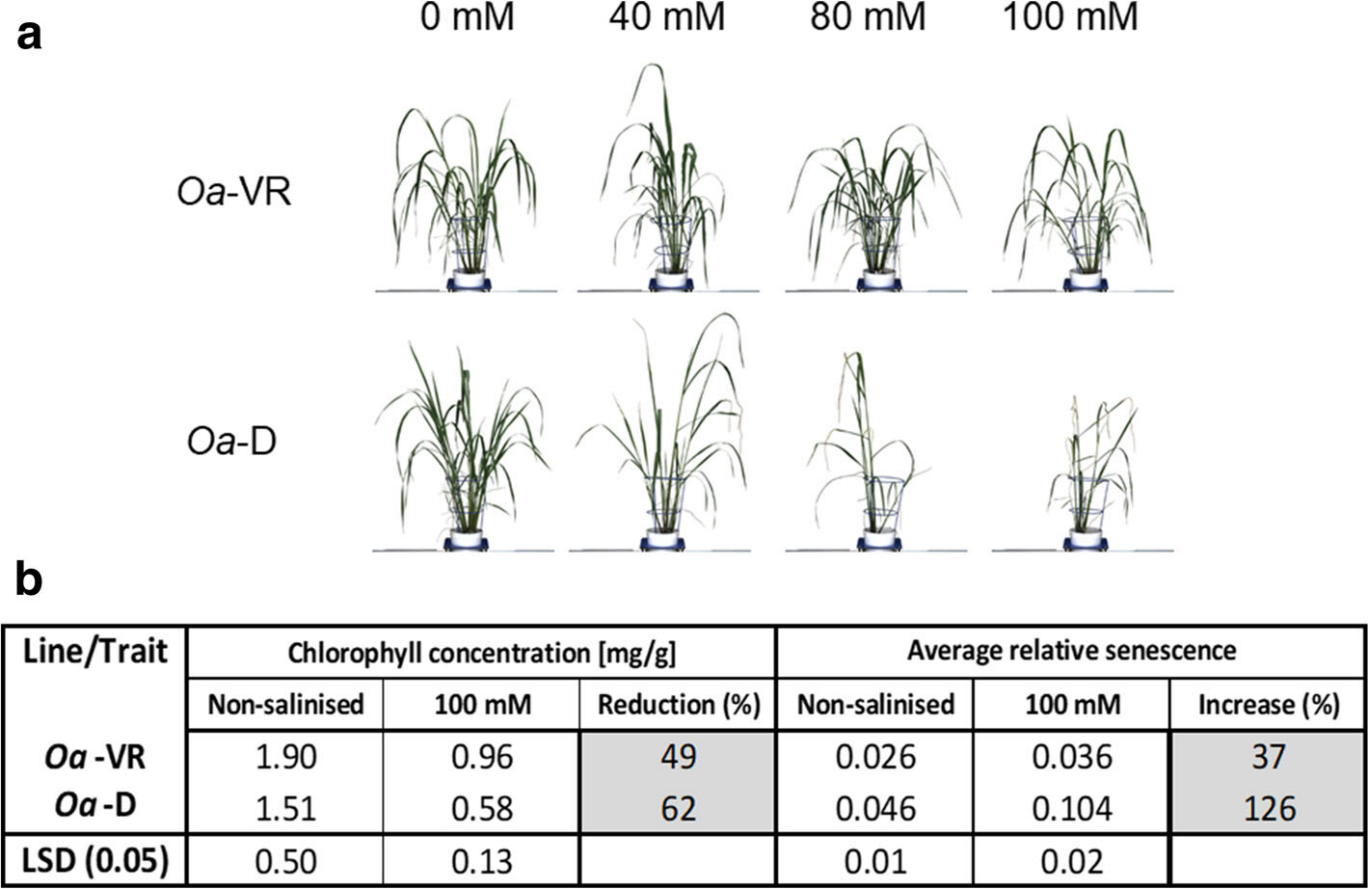
**a** Phenotypic changes in response to the different salt treatments 30 days after salting for the salt-tolerant *Oa*-VR and the salt-sensitive (*Oa*-D). **b** Chlorophyll concentration and average relative senescence under non-salinised (0 mM) and salinised (100 mM NaCl) treatments for both tested genotypes

Shoot images generated in the Plant Accelerator generated an estimate of *relative* leaf senescence using fluorescence optics, even though these values differ from visual analyses by SES, which showed that non-salinised leaves had not begun to senesce. However, the relative effects of NaCl on canopy development and the reported changes in senescence in salinised plants (Fig. 4) provide an accurate assessment of the impact of salt on *Oa*-VR and *Oa*-D (Hairmansis et al. 2014). Necrosis of older leaves was seen in the salt-sensitive genotype *Oa*-D after 30 d treatment with 40 mM NaCl, while the putatively salt-tolerant *Oa*-VR had minor leaf damage, even at 80 to 100 mM NaCl (Fig. 4). *Oa*-VR exhibited less than a 40% increase in relative senescence at 100 mM NaCl compared with the control, while an increase of more than 120% was recorded for *Oa*-D (Fig. 4). Furthermore, the impact of 100 mM NaCl on chlorophyll content was smaller in *Oa*-VR than in *Oa*-D (Fig. 4).

**Fig. 5:**
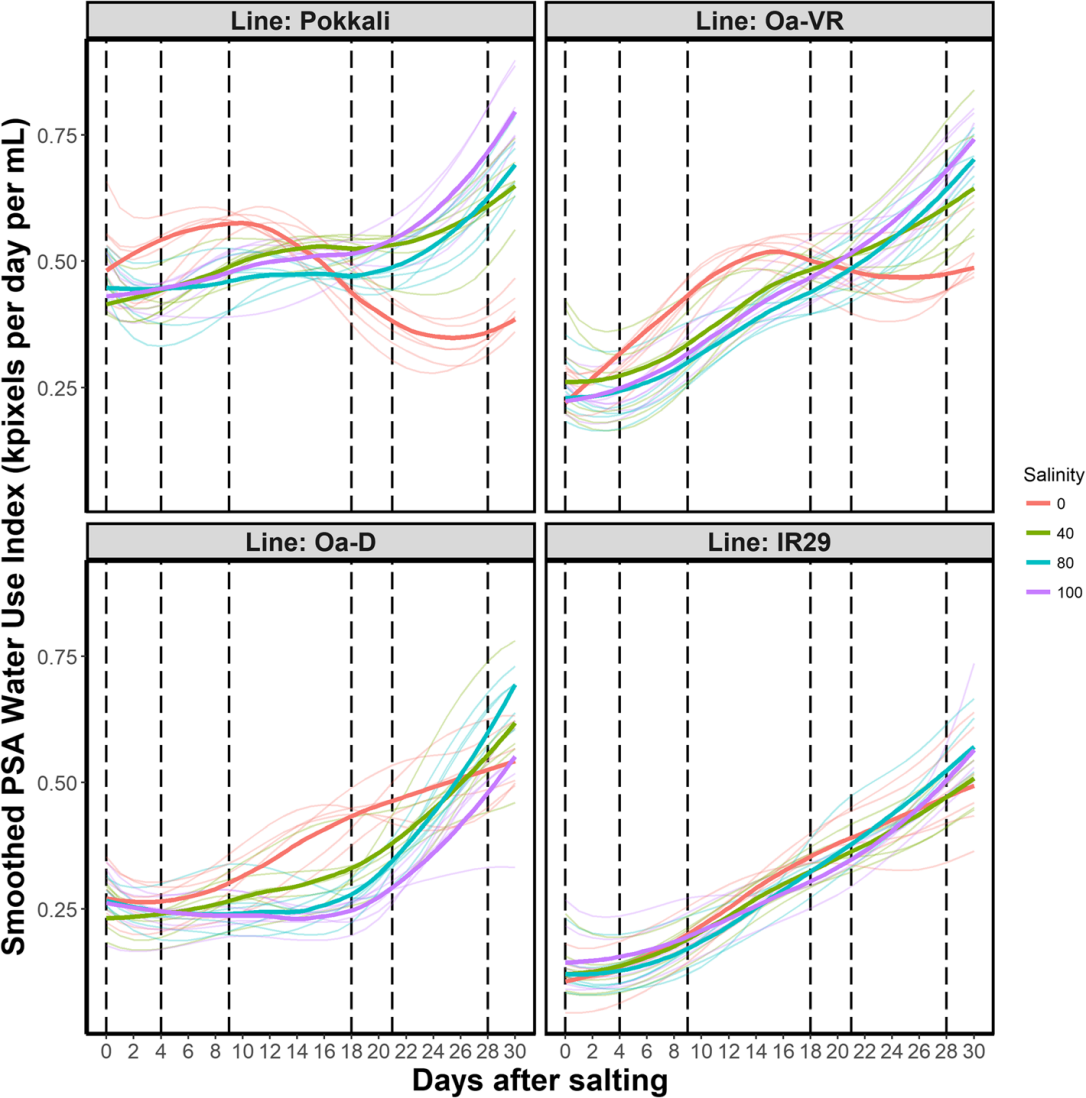
Relationship between growth and water use during salt treatment. Smoothed PSA Water Use Index is shown for the selected genotypes under salt treatments and non-salinised control conditions. The values were obtained by dividing the total increase in sPSA for each interval by the total water loss in the same interval. Thin lines represent individual plants. Bold lines represent the grand average of the six replicates for each treatment. Vertical broken lines represent the tested intervals

Compared with controls, WUI was impaired immediately after salt was applied (Fig. 5). While WUI continued to increase in *Oa*-VR throughout the experiment at all salt levels (in *Oa*-D at 80 and 100 mM NaCl), it accelerated only after 14 d of salt treatment. Control plants used water more efficiently than salt-treated plants up until 18 DAS and 24 DAS in *Oa*-VR and *Oa*-D, respectively. At 100 mM NaCl, *Oa*-VR used water substantially more efficiently than *Oa*-D, with WUI 25% higher at 100 mM NaCl by the end of the experiment in *Oa*-VR.

Both Pokkali and *Oa*-VR had a 36% lower fresh biomass under the higher salt treatment (100 mM NaCl) compared with non-salinised controls, while higher reductions were recorded for IR29 and *Oa*-D (49 and 53%, respectively; Additional file 7: Table S3).

## Discussion

Complementary approaches were taken to assess the salinity tolerance of lines/accessions of three rice species, *O. sativa*, *O. australiensis* and *O. meridionalis*. In a preliminary screening prior to these experiments, a survey of a wide range of wild *Oryza* accessions alongside Pokkali and IR29 produced a ‘short-list’ of five accessions chosen from *O. australiensis* and *O. meridionalis* that were selected for contrasting tolerance and sensitivity to salinity during early vegetative growth. The wild *Oryza* accessions chosen for this study evolved in geographically isolated populations, thereby broadening the range of genetic diversity and, with it, the opportunity to discover novel salt tolerance mechanisms (Menguer et al. 2017). However, the preliminary goal was to find contrasting salt tolerance within the same species in order to facilitate subsequent experiments involving mapping populations and comparative proteomics. In this paper, we report on one destructive experiment, with salt levels maintained at a steady state of 40 and 80 mM NaCl, and the second non-destructive experiment where soil was saturated initially with saline solution then followed by daily fresh water applications to replace evaporation and transpiration. The use of a series of images of plants in the Plant Accelerator gave a more dynamic picture of salinity tolerance than could be achieved by destructive measurements as in the first experiment. Ion concentrations in the YFL and phenotypic observations from the first experiment were seminal to developing a salt tolerance ranking.

Multiple strands of evidence from our data, including biomass, leaf visual symptoms, gas exchange and ion concentrations, confirm the wide range of tolerances to salt in the genotypes of wild and cultivated rice selected for these experiments. For example, chlorophyll levels were almost 50% lower in IR29 at 40 mM NaCl but were unaffected in *Oa*-VR, similar to contrasts in tolerance reported previously (Lutts et al. 1996), where 50 mM NaCl lowered chlorophyll levels by up to 70%. The criteria reported in Fig. 1 support the long-established view that Pokkali is highly tolerant to salt (Yeo et al. 1990) but make a case that the wild *O. australiensis* species (*Oa*-VR) has at least the same level of salt tolerance. In the first experiment, salt tolerance in *Oa*-VR was evident after 25 d of 80 mM NaCl, where shoot biomass was reduced by 58% in Pokkali compared with controls, while the reduction in biomass in *Oa*-VR was marginally less (50%). Moreover, symptoms of leaf damage in *Oa*-VR due to NaCl were significantly less pronounced than those seen in Pokkali.

The additional level of salt tolerance found in *Oa*-VR offers a potential tool for crop improvement, especially in that *Oa*-VR is from a wild *Oryza* population with the unique EE genome (Jacquemin et al. 2013), and is thus phylogenetically remote from *O. sativa*; this enhances the possibility of identifying novel mechanisms of salt tolerance unique to *O. australiensis*. By contrast, IR29 is reputedly highly salt-sensitive (Martinez-Atienza et al. 2006; Islam et al. 2011). Surprisingly, for the most salt-sensitive of the wild rice genotypes (*Oa*-D and *Oa*-KR) in very moderate salinity (40 mM NaCl), biomass and ion concentrations were more strongly affected by salt than leaf symptoms, possibly indicating genotypic variation in tissue tolerance to NaCl, as reported earlier (Yeo et al. 1990). In reverse, the very slow absolute growth rates of IR29 appeared, paradoxically, to result in a small effect of salt on relative growth rates (Fig. 3) but much larger effects on senescence (Fig. 1a). This suggests that a range of performance criteria is essential to distinguish the intrinsic differences in salt tolerances in screening experiments. This underlines the polygenic nature of salt tolerance, where genes determining ion import, compartmentation and metabolic responses to salt are likely to play a collective role in physiological tolerance (Munns et al. 2008). Therefore, based on the overall indicators of salt tolerance and rates of shoot development, *Oa*-VR and *Oa*-D were chosen as complementary *O. australiensis* genotypes for image analysis (Fig. 4), representing contrasting tolerance to salt in otherwise indistinguishable *O. australiensis* accessions. While the salt-tolerant genotype (*Oa*-VR) is from the Northern Territory and the salt-sensitive accession is from the Kimberley region of Western Australia, there is no obvious basis for predicting their respective tolerances to salinity without a fine-scale investigation of the collection sites and the seasonal fluctuations in soil water content and soil chemistry.

The rate at which shoot growth responded to salt (Experiment 2), as well as the internal Na^+^ and K^+^ concentrations of young leaves (Experiment 1), provide insights into possible mechanisms of tolerance. In rice, only part of the Na^+^ load reaching the leaves is taken up symplastically by the roots (Krishnamurthy et al. 2009), entering the transpiration stream and further regulated under the control of a suite of transporters. The low Na^+^/K^+^ ratios found in both *Oa*-VR and Pokkali (< 0.5) suggest that active mechanisms are in play to exclude Na^+^, even when the external solution was fixed at 80 mM NaCl for 30 d. Early clues as to how this is achieved came from a QTL (Ren et al. 2005), now known to contain the OsHKT1;5 gene, which enhances Na^+^ exclusion in rice (Hauser et al. 2010). Davenport et al. (2007) and others have established that the HKT1 transporters in *Arabidopsis* retrieve Na^+^ from the xylem. In general, high-affinity K^+^ uptake systems have now been shown to be pivotal for the management of salinity and deficiency symptoms in rice (Suzuki et al. 2016), as well as other species such as *Arabidopsis* and wheat (Byrt et al. 2007; Munns et al. 2008; Hauser et al. 2010). Further candidates such as the SOS1 transporter might also play a key part in the removal of Na^+^ from the xylem stream (Shi et al. 2002). The complexity of the rice HKT transporters identified in *O. sativa* (Garciadeblás et al. 2003) has not yet been explored in a wider range of *Oryza* genetic backgrounds. The levels of tolerance reported for *O. australiensis* should stimulate an analysis of the expression of genes regulating Na^+^ and K^+^ transport and the functional properties of these transporters, which may have evolved in lineages of geographically isolated communities from the Australian savannah.

Sodium exclusion appeared to operate effectively in Pokkali and *Oa*-VR but failed in other wild rice accessions where Na^+^/K^+^ exceeded 2.0 in the most severe cases at 80 mM NaCl. An earlier study reported leaf Na^+^/K^+^ ratios of 4.4 in 21 *indica* rice lines after 48 d of about 35 mM NaCl (Asch et al. 2000), reinforcing the view that *Oa*-VR is tolerant to salt. Supporting this claim, Na^+^ concentrations in Pokkali and *Oa*-VR calculated on a tissue-water basis were half those in the external solution when the roots were in an 80 mM solution. These contrasting degrees of Na^+^ exclusion and the consequences for plant performance are illustrated by the strong relationship between ST and the accumulation of Na^+^ (Fig. 2). Based on the observation that diminished apoplastic uptake of Na^+^ in the roots of Pokkali (Krishnamurthy et al. 2011) enhances Na^+^ exclusion, the degree of bypass flow in *Oa*-VR and the other genotypes in the current study is a priority for identifying the mechanism of salt tolerance. The consequences of Na^+^ loads in leaves for shoot physiology (SES, chlorophyll content, photosynthesis and tiller development) was apparent for the wild *Oryza* species as well as the two *O. sativa* standard genotypes, with strong correlations between ion levels and leaf damage.

In the second experiment, relative growth rates could be observed continuously and non-destructively, revealing an impact of salt even in the first 4 DAS (Additional file 5 Figure S3). A binary impact of salt on plants is exerted through osmotic stress and ion toxicity (Greenway and Munns 1980). The long-term impact of salt in this 30-d salt treatment was primarily due to toxic effects of Na^+^ rather than osmotic stress, which would have been most apparent in the earliest stages of the treatment period when tissue ion levels were lowest and osmotic adjustment was not yet established (Munns et al. 2016). The more salt-sensitive genotypes appeared to have less capacity to exclude salt, causing leaf Na^+^ and K^+^ concentrations to rise above parity and cause toxicity and metabolic impairment.

Water use efficiency was substantially greater in *Oa*-VR than *Oa*-D, particularly in the first two weeks after salt was applied, suggesting that the resilience of photosynthesis observed in salt-treated *Oa*-VR plants sustained growth (PSA) even as stomatal conductance fell by 60%. WUI values for *Oa*-D plants at 100 mM NaCl were notably lower than those at 40 and 80 mM NaCl, reflecting the progressively higher impact of NaCl on hydraulics in this sensitive genotype as concentrations increased from 40 to 100 mM NaCl. This trend of low WUI in salt-treated plants is consistent with previous studies of *indica* and *aus* rice (Al-Tamimi et al. 2016), as well as barley and wheat (Harris et al. 2010).

The effects of salt are dynamic, depending both upon relative growth rates and ion delivery and root:shoot ratios (Munns et al. 2016). Non-destructive measurements of growth showed that the relationship between control and salt-treated plants varied substantially over the time-course of treatment in all genotypes. This was partly due to the different developmental programs of each genotype, with Pokkali characterised by vigorous early growth and an early transition to flowering in non-saline conditions, when vegetative growth arrested; the transition to flowering was delayed in salt-treated plants. Such developmental effects are likely to be a factor in the impact of salinity on yield (Khatun et al. 1995). Among the wild rices, we have observed strong contrasts in photoperiod sensitivity between accessions, resulting in large differences in duration of vegetative growth. We speculate that this would affect the time-course of NaCl accumulation and its impact on biomass and grain yield.

Under paddy and rainfed conditions, salt levels in the root medium are unlikely to remain constant as they did in the treatment regime applied in the first experiment. This variation in salt load was better represented in the Plant Accelerator (Experiment 2), where soil was salinised and then transpired water replaced with fresh water to the soil surface daily. We contend that these contrasting regimes of salt application mimicked both steady-state and transient salinisation, including the salt loads imposed on rice paddies following spasmodic tidal surges. The ranking of salt-tolerance for both the *O. sativa* ‘standard’ genotypes and the four wild rice relatives was broadly maintained under the two experimental regimes we employed.

In this study, we explored the naturally occurring variation in salt tolerance among some of rice’s wild relatives in comparisons to selected *O. sativa* cultivars. Despite the substantial genetic distance between *O. australiensis* (taxon E) and *Oryza sativa* (taxon A), several studies have managed to leap this species barrier, allowing these two species to be crossed (Morinaga et al. 1960; Nezu et al. 1960). Another study reported a rapid phenotype recovery of the recurrent parent after only two backcrosses (Multani et al. 1994). Using this backcrossing approach, *O. australiensis* accessions have been used in breeding programs as a source of tolerance to biotic stresses including bacterial blight resistance (Brar and Khush 1997), brown planthopper resistance (Jena et al. 2006) and blast resistance (Jeung et al. 2007; Suh et al. 2009). Our study highlights the potential use of the Australian wild-species alleles in breeding programs to exploit variations in abiotic stress generally and salinity tolerance in particular. However, harnessing alleles from wild relatives of rice that confer salt tolerance and applying them to modern cultivars remains a long-term objective until mechanisms of tolerance become clearer.

## Additional files

Additional file 1: **FigureS1.** Relationships between Projected Shoot Area (kpixels) 28 and 30 days after salting with Fresh Weight and Dry Weight based on 168 individual plants using the fluorescence images. Squared Pearson correlation coefficients are given on the right. (PNG 152 kb)

Additional file 2: **Table S1.** Shoot dry weight, shoot fresh weight, chlorophyll concentration and photosynthetic rate for the four wild *Oryza* accessions and *O. sativa* controls. (PNG 15 kb)

Additional file 3: **Table S2.** Linear correlation (r values) between various physiological characteristics measured for the four wild *Oryza* accessions and *O. sativa* controls combined at seedling stage grown under 80 mM NaCl for 30 d. * = Significant at 5% level of probability and ** = Significant at 1% level of probability. (PNG 17 kb)

Additional file 4: **Figure 2.** Smoothed Projected Shoot Area (described by kpixels) of Absolute Growth Rates over six intervals within 0–28 days after salting. X-axis represents the salt levels and the error bars represent ±1/2 Confidence Interval. (PNG 85 kb)

Additional file 5: **Figure S3.** Smoothed Projected Shoot Area (described by kpixels) of Relative Growth Rates over the four salt treatments within 0–25 days after salting. Error bars represent ±1/2 Confidence Interval. (PNG 81 kb)

Additional file 6: **Figure S4.** Absolute growth rates of all tested genotypes from 0 to 30 DAS including non-salinised controls. Smoothed AGR values were derived from projected shoot area (PSA) values to which splines had been fitted. Thin lines represent individual plants. Bold lines represent the grand average of the six replicates plants for each treatment. The vertical broken lines represent the tested intervals. (PNG 357 kb)

Additional file 7: **Table S3.** Photosynthetic rate, stomatal conductance, number of tillers and shoot fresh weight of the four wild *Oryza* accessions and *O. sativa* controls. The first three traits were evaluated on 29 DAS while shoot fresh weight was measured on the termination of the experiment, on 30 DAS. Two measurements were excluded from the stomatal conductance analysis as they gave large negative values (− 30 and − 50). Reduction values were rounded to the nearest integer. (PNG 32 kb)

## Abbreviations

AGT: Absolute Growth Rate
ANOVA: Analysis of Variance
DAS: Days After Salting
DAT: Days After Transplanting
DF: Degrees of Freedom
EC: Electrical Conductivity
FLUO: Fluorescence
IRRI: International Rice Research Institute
PSA: Projected Shoot Area
PVC: Polyvinyl Chloride
QTL: Quantitative Trait Locus
RGB: Red-Green-Blue
RGR: Relative Growth Rate
SDW: Shoot Dry Weight
SES: Standard Evaluation System
SFW: Shoot Fresh Weight
sPSA: Smoothed Projected Shoot Area
ST: Salinity Tolerance
WUI: Water Use Index
YFL: Youngest Fully Expanded Leaf

## References

[CR1] Al-Tamimi N, Brien C, Oakey H (2016). Salinity tolerance loci revealed in rice using high-throughput non-invasive phenotyping. Nat Commun.

[CR2] Asch F, Dingkuhn M, Dörffling K, Miezan K (2000). Leaf K / Na ratio predicts salinity induced yield loss in irrigated rice. Euphytica.

[CR3] Atieno J, Li Y, Langridge P (2017). Exploring genetic variation for salinity tolerance in chickpea using image-based phenotyping. Sci Rep.

[CR4] Atwell BJ, Wang H, Scafaro AP (2014). Could abiotic stress tolerance in wild relatives of rice be used to improve *Oryza sativa*?. Plant Sci.

[CR5] Ballini E, Berruyer R, Morel JB (2007). Modern elite rice varieties of the “green revolution” have retained a large introgression from wild rice around the Pi33 rice blast resistance locus. New Phytol.

[CR6] Berger B, Bas De Regt MT (2012). High-throughput phenotyping in plants shoots. Methods Mol Biol.

[CR7] Brar DS, Khush GS (1997). Alien introgression in rice. Plant Mol Biol.

[CR8] Brien, C. J. (2018) dae: Functions useful in the design and ANOVA of experiments. Version 3.0-16

[CR9] Brozynska M, Copetti D, Furtado A (2016). Sequencing of Australian wild rice genomes reveals ancestral relationships with domesticated rice. Plant Biotech J.

[CR10] Butler DG, Cullis BR, Gilmour AR, Gogel BJ (2009) Analysis of Mixed Models for S language environments: ASReml-R reference manual. Brisbane, DPI Publications

[CR11] Byrt CS, Platten JD, Spielmeyer W (2007). HKT1;5-like cation transporters linked to Na^+^ exclusion loci in wheat, *Nax2* and *Kna1*. Plant Physiol.

[CR12] Campbell MT, Du Q, Liu K (2017) A comprehensive image-based phenomic analysis reveals the complex genetic architecture of shoot growth dynamics in rice. Plant Genome 10:2 10.3835/plantgenome2016.07.0064 28724075

[CR13] Campbell MT, Knecht AC, Berger B (2015) Integrating image-based phenomics and association analysis to dissect the genetic architecture of temporal salinity responses in rice. Plant Physiol 168:1476–1489 10.1104/pp.15.00450 PMC4528749 26111541

[CR14] Davenport RJ, Muñoz-Mayor A, Jha D (2007). The Na^+^ transporter AtHKT1;1 controls retrieval of Na^+^ from the xylem in Arabidopsis. Plant Cell Environ.

[CR15] Flowers TJ (2004). Improving crop salt tolerance. J Exp Bot.

[CR16] Fukuda A, Nakamura A, Tagiri A (2004) Function, intracellular localization and the importance in salt tolerance of a vacuolar Na^+^/H^+^ antiporter from rice. Plant Cell Physiol 45:146–159 10.1093/pcp/pch014 14988485

[CR17] Garciadeblás B, Senn ME, Bañuelos MA, Rodríguez-Navarro A (2003). Sodium transport and HKT transporters: the rice model. Plant J.

[CR18] Grattan SR, Shannon MC, Roberts SR (2002). Rice is more sensitive to salinity than previously thought. Calif Agric.

[CR19] Greenway H, Munns R (1980). Mechanisms of salt tolerance in nonhalophytes. Annu Rev Plant Biol.

[CR20] Gregorio GB, Senadhira D (1993). Genetic analysis of salinity tolerance in rice (*Oryza sativa* L.). Theor Appl Genet.

[CR21] Hairmansis A, Berger B, Tester M, Roy SJ (2014). Image-based phenotyping for non-destructive screening of different salinity tolerance traits in rice. Rice.

[CR22] Harris BN, Sadras VO, Tester M (2010). A water-centred framework to assess the effects of salinity on the growth and yield of wheat and barley. Plant Soil.

[CR23] Hauser F, Horie T (2010). A conserved primary salt tolerance mechanism mediated by HKT transporters: a mechanism for sodium exclusion and maintenance of high K^+^/Na^+^ ratio in leaves during salinity stress. Plant Cell Environ.

[CR24] Henry RJ, Rice N, Waters DLE (2010). *Australian Oryza*: utility and conservation. Rice.

[CR25] IRRI (2013). Standard Evaluation System (SES) for Rice.

[CR26] Islam MR, Salam MA, Hassan L, Collard BCY, Singh RK, Gregorio GB (2011). QTL mapping for salinity tolerance in rice. Physiol Mol Biol Plants.

[CR27] Ismail AM, Horie T (2017). Molecular breeding approaches for improving salt tolerance. Annu Rev Plant Biol.

[CR28] Jacquemin J, Bhatia D, Singh K, Wing RA (2013). The international *Oryza* map alignment project: development of a genus-wide comparative genomics platform to help solve the 9 billion-people question. Curr Opin Plant Biol.

[CR29] Jena KK, Jeung JU, Lee JH (2006). High-resolution mapping of a new brown planthopper (BPH) resistance gene, *Bph18*(t), and marker-assisted selection for BPH resistance in rice (*Oryza sativa* L.). Theor Appl Genet.

[CR30] Jeung JU, Kim BR, Cho YC (2007). A novel gene, *Pi40*(t), linked to the DNA markers derived from NBS-LRR motifs confers broad spectrum of blast resistance in rice. Theor Appl Genet.

[CR31] Khatun S, Flowers TJ (1995). Effects of salinity on seed set in rice. Plant Cell Environ.

[CR32] Khush GS (1997) Origin, dispersal, cultivation and variation of rice. Plant Mol Biol 35:25–34 9291957

[CR33] Khush GS (2005) What it will take to feed 5.0 billion rice consumers in 2030. Plant Mol Biol 59(1):–6 10.1007/s11103-005-2159-5 16217597

[CR34] Krishnamurthy P, Ranathunge K, Franke R (2009). The role of root apoplastic transport barriers in salt tolerance of rice (*Oryza sativa* L.). Planta.

[CR35] Krishnamurthy P, Ranathunge K, Nayak S (2011). Root apoplastic barriers block Na^+^ transport to shoots in rice (*Oryza sativa* L.). J Exp Bot.

[CR36] Lang N, Li Z, Buu B (2001). Microsatellite markers linked to salt tolerance in rice. Omonrice.

[CR37] Lutts S, Kinet JM, Bouharmont J (1995). Changes in plant response to NaCl during development of rice (*Oryza sativa* L.) varieties differing in salinity resistance. J Exp Bot.

[CR38] Lutts S, Kinet JM, Bouharmont J (1996) NaCl-induced senescence in leaves of rice (*Oryza sativa* L.) cultivars differing in salinity resistance. Ann Bot 78:389–398

[CR39] Mackinney G (1941). Absorption of light by chlorophyll solutions. J Biol Chem.

[CR40] Martinez-Atienza J, Jiang X, Garciadeblas B (2006) Conservation of the salt overly sensitive pathway in rice. Plant Physiol 143:1001–1012 10.1104/pp.106.092635 PMC1803719 17142477

[CR41] Menguer PK, Sperotto RA, Ricachenevsky FK (2017). A walk on the wild side : *Oryza* species as source for rice abiotic stress tolerance. Genet Mol Biol.

[CR42] Morinaga T, Kuriyama H (1960). Interspecific hybrids and genomic constitution of various species in the genus *Oryza*. Agric Hortic.

[CR43] Multani DS, Jena KK, Brar DS, de los Reyes BG, Angeles ER, Khush GS (1994) Development of monosomic alien addition lines and introgression of genes from *Oryza australiensis* Domin. to cultivated rice *O. sativa* L. Theor Appl Genet 88:102–109 10.1007/BF00222401 24185889

[CR44] Munns R, James RA, Gilliham M (2016). Tissue tolerance: an essential but elusive trait for salt-tolerant crops. Funct Plant Biol.

[CR45] Munns R, Tester M (2008). Mechanisms of salinity tolerance. Annu Rev Plant Biol.

[CR46] Nezu M, Katayama TC, Kihara H (1960). Genetic study of the genus *Oryza*. I. Crossability and chromosomal affinity among 17 species. Seiken Jiho.

[CR47] Ochiai K, Matoh T (2002). Characterization of the Na^+^ delivery from roots to shoots in rice under saline stress: excessive salt enhances apoplastic transport in rice plants. Soil Sci Plant Nutr.

[CR48] Qadir M, Quillérou E, Nangia V (2014). Economics of salt-induced land degradation and restoration. Nat Resour Forum.

[CR49] R Core Team (2018) R: A language and environment for statistical computing. Vienna, Austria: R Foundation for Statistical Computing.

[CR50] Rahman ML, Jiang W, Chu SH (2009). High-resolution mapping of two rice brown planthopper resistance genes, *Bph20*(t) and *Bph21*(t), originating from *Oryza minuta*. Theor Appl Genet.

[CR51] Ren Z-H, Gao J-P, Li L (2005). A rice quantitative trait locus for salt tolerance encodes a sodium transporter. Nat Genet.

[CR52] Sabouri H, Sabouri A (2008). New evidence of QTLs attributed to salinity tolerance in. African J Biotechnol.

[CR53] Scafaro AP, Gallé A, Van Rie J (2016). Heat tolerance in a wild *Oryza* species is attributed to maintenance of rubisco activation by a thermally stable rubisco activase ortholog. New Phytol.

[CR54] Scafaro AP, Haynes PA, Atwell BJ (2010). Physiological and molecular changes in *Oryza meridionalis* ng., a heat-tolerant species of wild rice. J Exp Bot.

[CR55] Shi H, Quintero FJ, Pardo JM, Zhu JK (2002). The putative plasma membrane Na^+^/H^+^ antiporter SOS1 controls long-distance Na^+^ transport in plants. Plant Cell.

[CR56] Stein JC, Yu Y, Copetti D (2018). Genomes of 13 domesticated and wild rice relatives highlight genetic conservation, turnover and innovation across the genus *Oryza*. Nat Genet.

[CR57] Suh JP, Roh JH, Cho YC (2009). The pi40 gene for durable resistance to rice blast and molecular analysis of pi40-advanced backcross breeding lines. Phytopathology.

[CR58] Suzuki K, Costa A, Nakayama H (2016). OsHKT2;2/1-mediated Na^+^ influx over K^+^ uptake in roots potentially increases toxic Na^+^ accumulation in a salt-tolerant landrace of rice Nona Bokra upon salinity stress. J Plant Res.

[CR59] Takagi H, Tamiru M, Abe A (2015). MutMap accelerates breeding of a salt-tolerant rice cultivar. Nat Biotechnol.

[CR60] Thomson MJ, de Ocampo M, Egdane J (2010) Characterizing the *Saltol* quantitative trait locus for salinity tolerance in rice. Rice 3:148–160

[CR61] Wang W, Vinocur B, Altman A (2003). Plant responses to drought, salinity and extreme temperatures: towards genetic engineering for stress tolerance. Planta.

[CR62] Wickham H (2009) ggplot2: Create Elegant Data Visualisations Using the Grammar of Graphics. R package version 2.2.1.

[CR63] Yadav R, Flowers TJ, Yeo A (1996). The involvement of the transpirational bypass flow in sodium uptake by high- and low-sodium-transporting lines of rice developed through intravarietal selection. Plant Cell Environ.

[CR64] Yao MZ, Wang JF, Chen HY, Zha HQ, Zhang HS (2005). Inheritance and QTL mapping of salt tolerance in rice. Rice Sci.

[CR65] Yeo AR, Yeo ME, Flowers SA, Flowers TJ (1990). Screening of rice (*Oryza sativa* L.) genotypes for physiological characters contributing to salinity resistance, and their relationship to overall performance. Theor Appl Genet.

[CR66] Yeo AR, Yeo ME, Flowers TJ (1987). The contribution of an apoplastic pathway to sodium uptake by rice roots in saline conditions. J Exp Bot.

[CR67] Zeng L, Shannon MC, Grieve CM (2002) Evaluation of salt tolerance in rice genotypes by multiple agronomic parameters. Euphytica:235–245

[CR68] Zhu Q, Zheng X, Luo J (2007). Multilocus analysis of nucleotide variation of *Oryza sativa* and its wild relatives: severe bottleneck during domestication of rice. Mol Biol Evol.

