# Supplementary figures and images for: Salinity tolerance in Australian wild Oryza species varies widely and matches that observed in O. sativa
Source: Rice (N Y). 2018 Dec 22;11:66. doi: 10.1186/s12284-018-0257-7 (PMC6303227; doi:10.1186/s12284-018-0257-7)

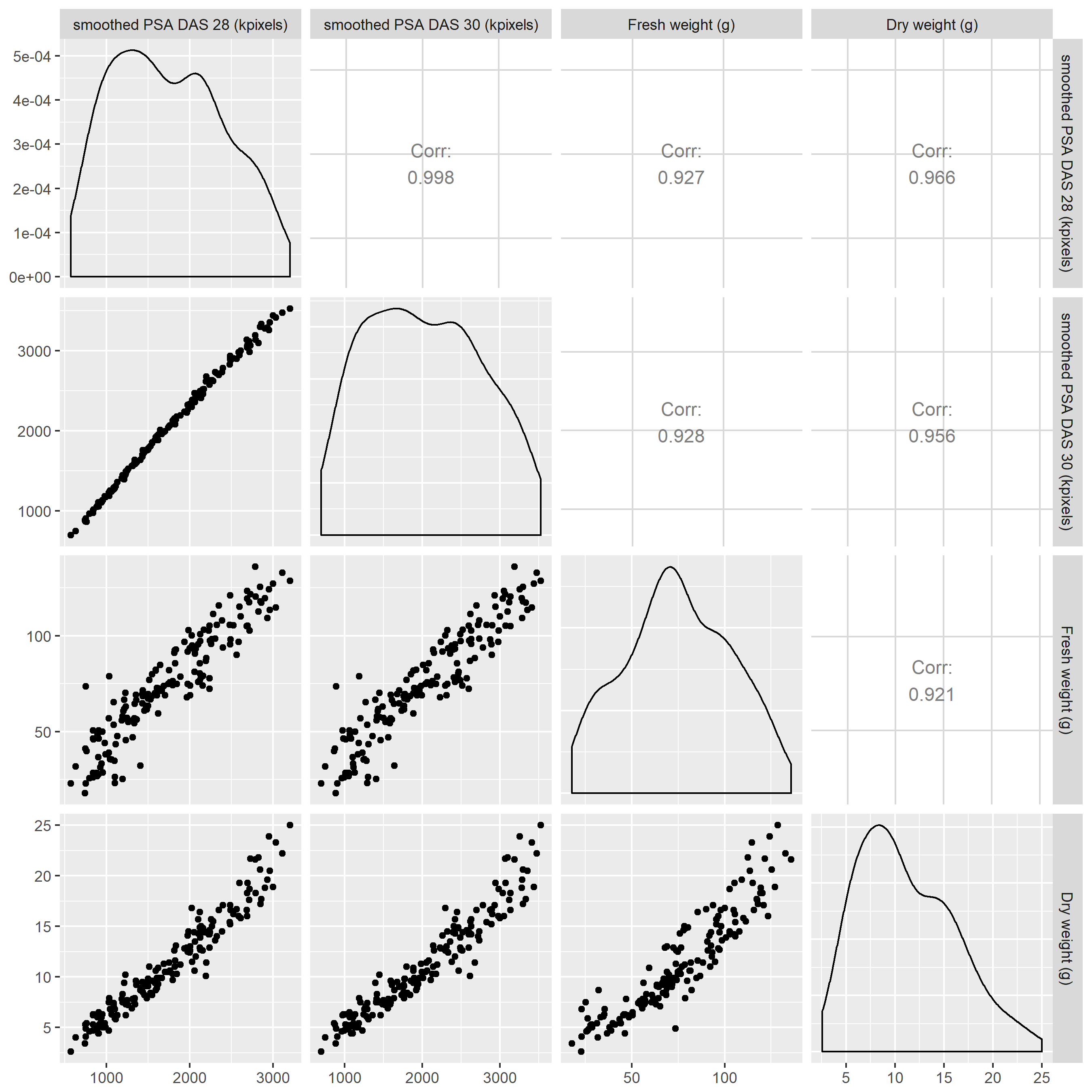

Supplement: Supplementary file 1 — FigureS1. Relationships between Projected Shoot Area (kpixels) 28 and 30 days after salting with Fresh Weight and Dry Weight based on 168 individual plants using the fluorescence images. Squared Pearson correlation coefficients are given on the right. (PNG 152 kb) [file 12284_2018_257_MOESM1_ESM.png]

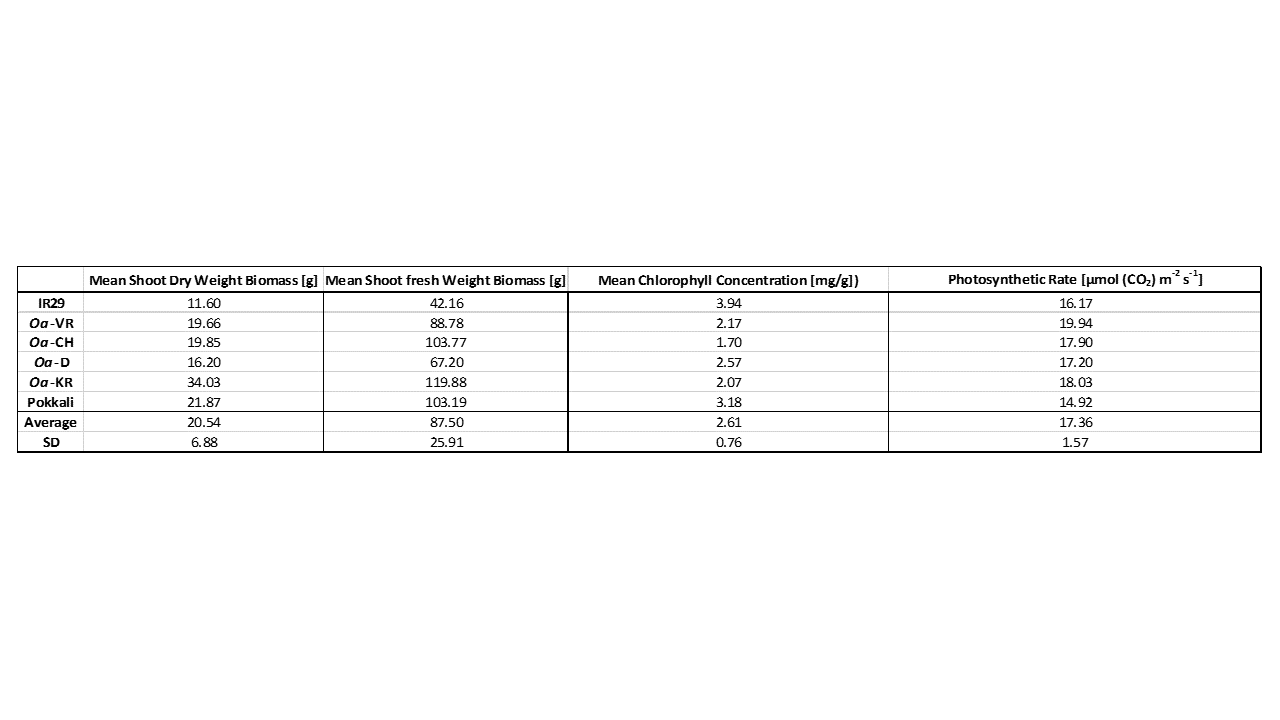

Supplement: Supplementary file 2 — Table S1. Shoot dry weight, shoot fresh weight, chlorophyll concentration and photosynthetic rate for the four wild Oryza accessions and O. sativa controls. (PNG 15 kb) [file 12284_2018_257_MOESM2_ESM.png]

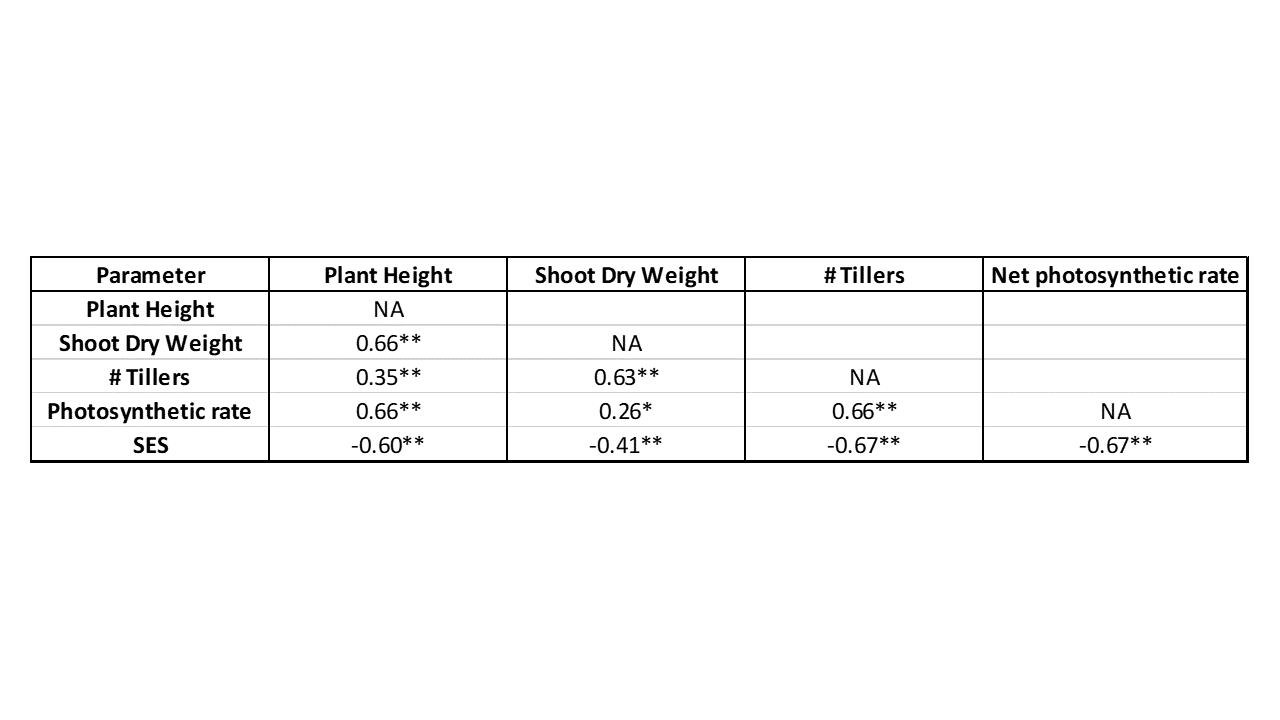

Supplement: Supplementary file 3 — Table S2. Linear correlation (r values) between various physiological characteristics measured for the four wild Oryza accessions and O. sativa controls combined at seedling stage grown under 80 mM NaCl for 30 d. * = Significant at 5% level of probability and ** = Significant at 1% level of probability. (PNG 17 kb) [file 12284_2018_257_MOESM3_ESM.png]

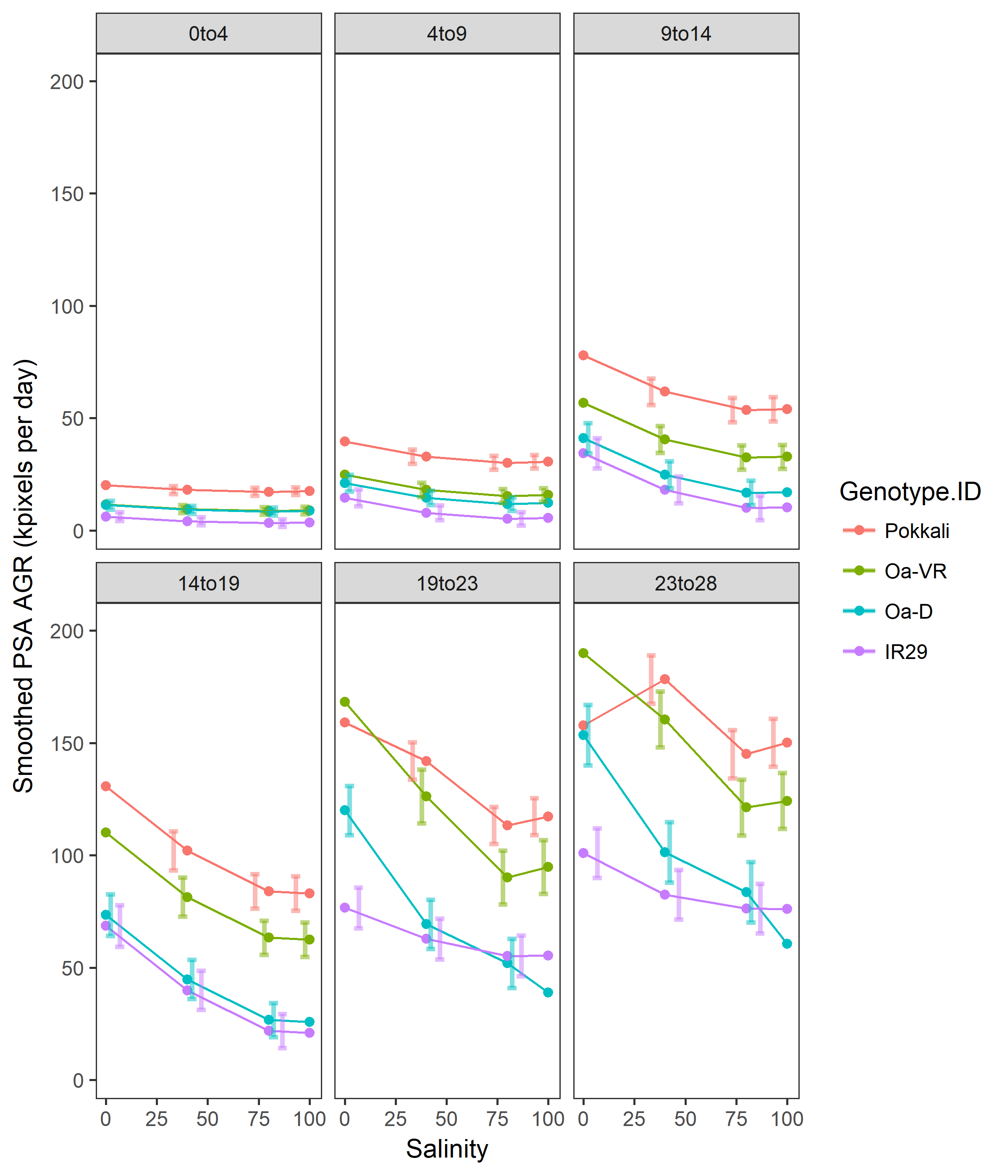

Supplement: Supplementary file 4 — Figure 2. Smoothed Projected Shoot Area (described by kpixels) of Absolute Growth Rates over six intervals within 0–28 days after salting. X-axis represents the salt levels and the error bars represent ±1/2 Confidence Interval. (PNG 85 kb) [file 12284_2018_257_MOESM4_ESM.png]

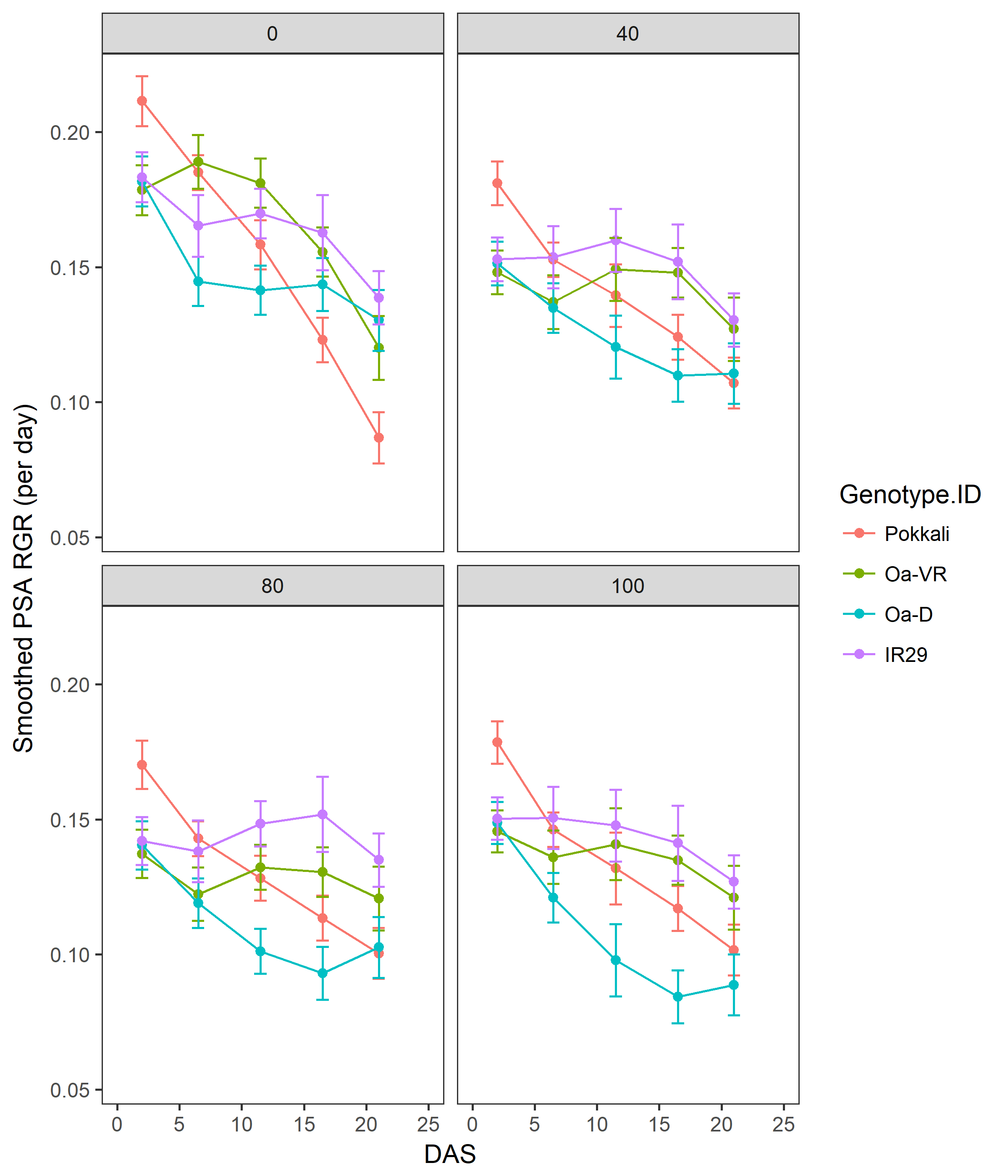

Supplement: Supplementary file 5 — Figure S3. Smoothed Projected Shoot Area (described by kpixels) of Relative Growth Rates over the four salt treatments within 0–25 days after salting. Error bars represent ±1/2 Confidence Interval. (PNG 81 kb) [file 12284_2018_257_MOESM5_ESM.png]

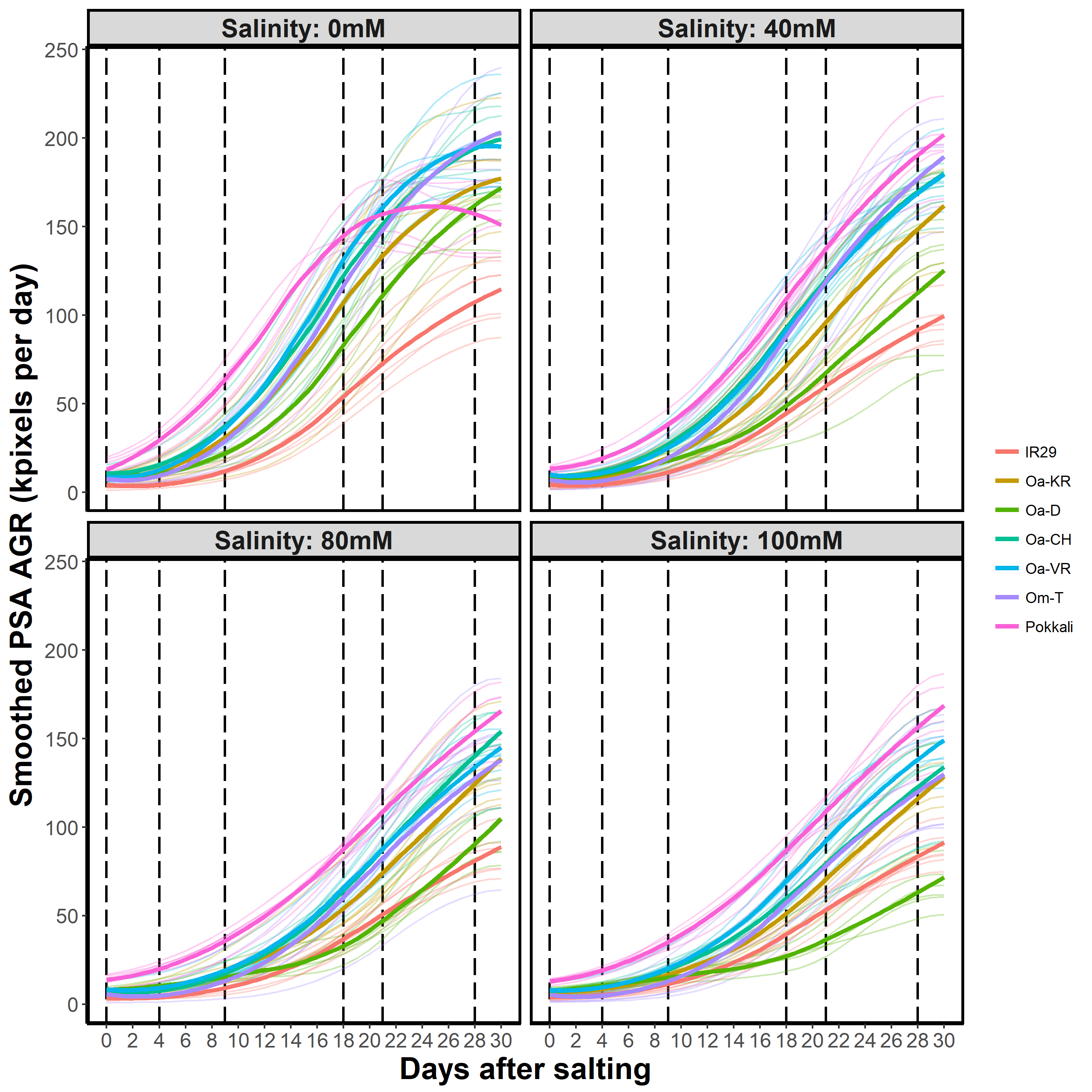

Supplement: Supplementary file 6 — Figure S4. Absolute growth rates of all tested genotypes from 0 to 30 DAS including non-salinised controls. Smoothed AGR values were derived from projected shoot area (PSA) values to which splines had been fitted. Thin lines represent individual plants. Bold lines represent the grand average of the six replicates plants for each treatment. The vertical broken lines represent the tested intervals. (PNG 357 kb) [file 12284_2018_257_MOESM6_ESM.png]

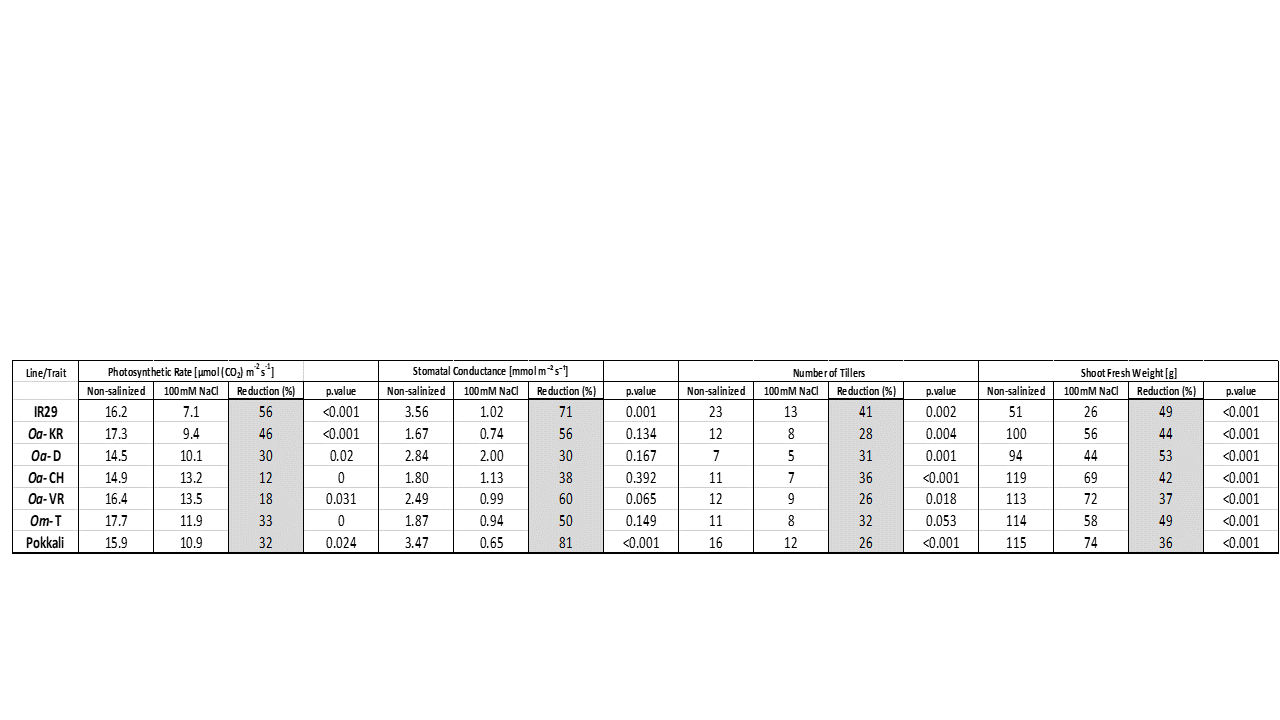

Supplement: Supplementary file 7 — Table S3. Photosynthetic rate, stomatal conductance, number of tillers and shoot fresh weight of the four wild Oryza accessions and O. sativa controls. The first three traits were evaluated on 29 DAS while shoot fresh weight was measured on the termination of the experiment, on 30 DAS. Two measurements were excluded from the stomatal conductance analysis as they gave large negative values (− 30 and − 50). Reduction values were rounded to the nearest integer. (PNG 32 kb) [file 12284_2018_257_MOESM7_ESM.png]
